# Impact of the Eras Protocols on Costs and Benefits in Two Italian Second-Level Healthcare Centers According to the National Waiting List Management Plan (PNGLA)

**DOI:** 10.3390/jcm15010347

**Published:** 2026-01-02

**Authors:** Francesco Frattini, Manrica Fabbi, Laura Bardelli, Federica Galli, Domenico Iovino, Linda Liepa, Marika Sharmayne Milani, Vincenzo Pappalardo, Franco Pavesi, Michele Surace, Luca Donnini, Diego Baù, Giovanni Poggialini, Paolo Covacich, Lorenzo Isella, Stefano Rausei

**Affiliations:** 1 Department of Surgery, Cittiglio-Angera Hospital, ASST Settelaghi, 21100 Varese, Italy; 2 Department of Biotechnology and Life Sciences (DBSV), Insubria University, 21100 Varese, Italy; 3 Orthopedics and Trauma Unit, Cittiglio-Angera Hospital, ASST Sette Laghi, 21100 Varese, Italy; 4 Department of Operations Management, ASST Sette Laghi, 21100 Varese, Italy

**Keywords:** ERAS, colorectal surgery, hospitalization, healthcare

## Abstract

**Background/Objectives**: To analyze the financial impact of the ERAS program in two major surgical procedures (colon resection for cancer and hip replacement) in two second-level healthcare centers. **Methods**: A cost–benefit analysis was carried out on four hypothetical scenarios, based on the rate of compliance with the ERAS program, focusing on the additional costs and the additional benefits deriving from the decrease in hospital stay caused by the application of the ERAS protocol, with particular regard to the interventions envisaged by the National Waiting List Management Plan (PNGLA). **Results**: In the most optimistic scenario, with a coefficient of application of ERAS of 100% and a number of 800 days of hospitalization gained per year, the revenue–cost ratio was equal to 2.92. In the least favorable scenario, with a coefficient of application of ERAS of 50% and a number of 400 days of hospitalization gained per year, the revenue–cost ratio was equal to 1.11. **Conclusions**: In all the scenarios, the revenue–cost ratio was higher than 1. Implementation of the ERAS program is feasible also in second-level centers with the costs for additional healthcare professionals. Application of the ERAS program leads to a more sustainable health policy with an improvement in the number of treated patients per year and an advantage in the waiting list.

## 1. Introduction

Enhanced recovery after surgery (ERAS) programs are a set of evidence-based practices spanning the perioperative period, designed to optimize postoperative outcomes by minimizing the physiological stress response and maintaining or rapidly restoring baseline function [1]. The concept that a structured, multimodal, multidisciplinary pathway can result in accelerated recovery after surgery first emerged in the early 2000s amongst patients undergoing colorectal surgery [2,3,4]. ERAS programs are now well-established in colorectal surgery, with the benefits over traditional care demonstrated by several meta-analyses of randomized controlled trials [5,6,7,8]. The first ERAS guidelines in colorectal surgery were published in 2005 [9]. Since the publication of the first ERAS colorectal guideline, a total of three subsequent sets of guidelines have been published. The current 2025 guideline reflects the collaborative efforts of the ERAS Society [10]. When comparing ERAS protocols with traditional care in colorectal surgery, ERAS significantly reduces the risk for postoperative morbidity and median length of stay (LOS) in the hospital.

ERAS is a multimodal approach optimizing the preoperative phase, aiming for minimally invasive surgery and the enhanced management of anesthesia during the intraoperative phase, and culminating in a proactive approach to postoperative recovery. These phases have specific costs relating to staff, the operating room, hospital facilities, devices, medicines, services, and LOS.

Increased LOS after surgery leads to the consumption of limited hospital resources and can result in bed-blocking and delays in care for other patients and impaired patient flow in the hospital.

Actually, even if the main results of ERAS application are related to patients’ safety, one of the most direct implications of improved patient recovery is a substantially reduced LOS. Generally, it also represents the simplest measure to verify ERAS effects.

Various meta-analyses have consistently demonstrated that ERAS decreases the postoperative LOS by an average of two days, as compared to standard perioperative care [6,11,12,13]. There is increasing recognition that same-day discharge (SDD), defined as hospital discharge within 24 h of surgery, or “hyper-ERAS”, may be feasible for a selected group of physiologically fit patients. In this context, the application of telemedicine can effectively support the patients’ follow-up after hospital discharge. Several reviews demonstrated that it is a feasible and effective communication technology alternative to face-to-face care [14,15]. Hence, telemedicine is proving to be a useful technology in the ERAS program.

It is important to point out that the healthcare system in Italy is still partially recovering from the coronavirus disease 2019 (COVID-19) pandemic. Italy was the first European country to suffer a strong impact, in particular in two northern regions, Lombardy and Veneto. A reorganization and dynamic distribution of national and regional healthcare resources were necessary to define algorithms for the management of patients requiring non-elective surgical procedures. This reorganization led to an increase in waiting times for surgical admission.

After the pandemic, in order to optimize the management of the waiting list of patients, the Italian Ministry of Health established the National Waiting List Management Plan (PNGLA, “Piano nazionale di gestione delle liste d’attesa”), approved in February 2019 as a State-Regions Statement. This plan aims at improving access to healthcare services and reducing waiting times, especially for services included in waiting lists, both outpatient and inpatient.

In detail, the PNGLA defines maximum waiting times for various services, divided into priority classes (urgent, short, deferrable, and plannable), establishes guidelines for regions and autonomous provinces for managing waiting lists, introduces monitoring and evaluation tools to verify the effectiveness of the measures adopted, promotes appropriate prescriptions and good clinical practices, involves various stakeholders in the healthcare system, including doctors, patients, pharmacies. The primary objective of the PNGLA is to ensure equitable and timely access to healthcare services, reducing inconvenience for citizens.

The aim of this study is to estimate the impact of the ERAS program on costs and benefits in two second-level centers for selected surgical procedures according to the PNGLA objectives.

## 2. Methods

A simulation of the costs and benefits deriving from the application of the ERAS protocol was conducted in collaboration with the Operations Management Department of ASST Sette Laghi.

As for the costs, preoperative and postoperative costs were identified as additional staff (1 nurse, 1 physiotherapist, and 1 nutritionist) and as the acquisition of a telemedicine platform.

Here, we report in detail the items of the ERAS protocol we apply in colorectal surgery. The ERAS program is divided into the following three phases: preadmission, intraoperative phase, and postoperative phase.

A key phase of ERAS protocols is preadmission, defined as the period of time between the patient’s admission and the surgery itself. The objectives of this phase are as follows: optimizing therapies, correcting any unhealthy habits and deficiencies (such as anemia), and educating the patient to achieve the best performance status (PS possible before surgery, promoting a more rapid postoperative recovery, a reduction in short- and long-term complications, and shorter hospital stays. This phase has proven essential for reducing the anxiety generated by waiting for surgery, postoperative pain, and, consequently, LOS. The process involves multidisciplinary discussions with the nurse, anesthesiologist, surgeon, dietitian, and physical therapist, with the aim of providing the patients with all the information they need and ensuring their full cooperation in developing their treatment plan.

A comprehensive multidisciplinary assessment will be performed, including blood tests, diagnostic imaging tests such as ECG and chest X-ray, and other staging tests in the case of cancer (if not previously performed). Finally, a surgical and anesthesiological reassessment will be performed, and, if necessary, consultations with other specialists identified by the attending physician will be sought.

Preoperative counseling in colon surgery is essential for the patient’s psychological preparation, education regarding the procedure, and, therefore, improved postoperative outcomes.

Approximately two weeks after the pre-admission assessment, the patient will have a counseling session. This meeting will take place in the presence of the surgeon, a designated nurse, the physiotherapist, and the patient’s caregiver.

The nurse will explain the nursing procedures that will be performed before the surgery. If necessary, the equipment will be explained, and contact information will be provided for the management of a stoma, should it be created during the surgical procedure.

The physiotherapist will reevaluate the patient after the first two weeks of physiotherapy preparation, possibly modifying the exercise recommendations.

The surgeon will clarify any doubts or concerns that may arise after the pre-admission assessment and while reading the brochure, especially regarding the specific techniques of the operation to which the patient will be subjected, reassessing the risks and benefits, and the management of possible complications.

In the intraoperative phase, the patient is identified after access in the operating room using a wristband and targeted questions to verify identity, allergies, and the surgical treatment for which the patient is a candidate.

The patient is prepared in the pre-operation room with identity verification, vascular access placement, positioning of the patient on the operating table, and application and positioning of anesthesia monitoring devices.

After execution of the pre-operative time-out by the anesthesiologist and surgeon, general anesthesia is administered as defined during pre-admission. A urinary catheter is placed after the patient has been intubated.

All the colon resections are performed in minimally invasive surgery. The nasogastric tube is removed at the end of the procedure.

On the first postoperative day, thromboprophylaxis is administered according to the Caprini Score: mechanical (elastic compression stockings) and/or administration of low molecular weight heparin. Fluid is reintroduced by mouth at least 2 h after awakening from anesthesia. Nutrition is resumed by mouth at least 6 h after awakening from anesthesia. Patients are mobilized in a chair, and physiotherapists control the patients to ensure correct breathing exercises are performed.

On the second postoperative day, infusion therapy is discontinued except in cases of protracted postoperative nausea and vomiting or hypotension. The bladder catheter is removed if urine output is satisfactory within 24 h, and spontaneous urination is monitored. Early refeeding continues with the diet planned for day 1 and fluid intake of at least 2 L.

The patient is maintained upright for at least eight hours a day. Ambulation is encouraged. Drainage and passage of gas or stool are monitored. Patients are educated in cases of enterostomy.

On the third postoperative day, a free diet is administered. The patients have free mobilization. Oral pain therapy is established to be continued at home if necessary. Health education for patients and caregivers is performed prior to discharge.

Starting on day 3, the patient may be considered ready for discharge. Items for safe discharge of the patients included the following:

- Patient autonomous in its functions;
- Stable vital signs;
- No signs or symptoms of complications;
- Independent ambulation;
- Well-tolerated solid diet;
- Completed gas channeling;
- Spontaneous diuresis;
- Absence of nausea, dizziness, or vomiting;
- Pain well controlled (NRS < 4) with oral therapy.

After the discharge, the patient is monitored at home using telemedicine.

Fifteen days after discharge, the patient will be emailed a Customer Satisfaction Report to assess their level of satisfaction with the ERAS protocol.

Informed consent was obtained from all subjects involved in the study for the application of the ERAS protocol.

The benefits were estimated as the revenues derived from the increase in the number of hospitalizations per year caused by the higher turnover of beds that the implementation of the ERAS protocol generates. Therefore, revenues were calculated through the following approach:

(1) estimation of the annual number of additional days of hospitalization;
(2) calculation of the number of new hospitalizations on the additional days (according to the case mix);
(3) calculation of the revenues deriving from additional hospitalizations per year.

The analysis was conducted on the volumes of four operating units in two second-level (spoke) hospitals: the General Surgery Unit and the Orthopedic Unit of Cittiglio Hospital (Varese, Italy) and the General Surgery Unit and the Orthopedic Unit of Angera Hospital (Varese, Italy).

For the estimation of the annual number of additional days of hospitalization, it was assumed that the ERAS protocol was applied only to the “major” interventions specific to general and orthopedic surgery included among the interventions of PNGLA, i.e., interventions for malignant colon tumors and hip replacement.

The analysis focused on operational efficiency, not taking into account the variation in case of complications.

The following four scenarios were considered:

- Scenario A: implementation of the ERAS protocol in 100% of cases considered and estimation of the resulting new hospitalizations based on the whole case mix of the units involved in the study;
- Scenario B: implementation of the ERAS protocol in 100% of cases considered and estimation of the resulting new hospitalizations based only on the PNGLA interventions performed by the units involved in the study;
- Scenario C: implementation of the ERAS protocol in 50% of cases considered and estimation of the resulting new hospitalizations based on the whole case mix of the units involved in the study;
- Scenario D: implementation of the ERAS protocol in 50% of cases considered and estimation of the resulting new hospitalizations based only on the PNGLA interventions performed by the units involved in the study.

In all the hypothetical scenarios, according to the literature [12,13,16,17], a 50% reduction in average hospital stay was estimated with the application of the ERAS protocol.

## 3. Results

The annual costs estimated for application of ERAS related to the procedures of colonic resection for malignant tumors and for hip replacement included:

- 120.000 euros per year for additional healthcare figures (1 nurse, 1 physiotherapist, and 1 nutritionist);
- 163.000 euros for a telemedicine platform for patients’ follow-up for the first year.

From September 2022 to October 2023, 37 colon resections for malignant tumors and 150 hip replacements were registered, with a mean postoperative stay of 8.43 days for colon resection and 8.59 days for hip replacement. By application of ERAS, the 1 year overall LOS for all the procedures of colonic resection could decrease from 312 days to 156 days, and for hip replacement from 1.289 to 644.5 days, respectively (Table 1).

Considering an implementation coefficient of the ERAS protocol at 100% (scenarios A and B), a total number of 800.5 gained days of hospitalization per year in general surgery and orthopedic units was estimated, equal to 151 additional annual hospitalizations considering all the procedures (scenario A) and 197 hospitalizations if only PNGLA procedures were considered (scenario B). It was possible to estimate an increase in patients treated per week ranging from +3.43 (scenario A) to +4.48 (scenario B).

Considering an implementation coefficient of the ERAS protocol at 50% (scenarios C and D), a total number of 400.25 gained days of hospitalization per year was estimated, equal to 65 additional annual hospitalizations considering all the procedures (scenario C) and 94 hospitalizations if only PNGLA procedures were considered (scenario D). It was possible to estimate an increase in patients treated per week ranging from +1.48 (scenario C) to +2.14 (scenario D).

The revenue–cost ratio was always higher than 1 in all four scenarios, ranging from 1.11 in scenario C to 2.92 in scenario B (Table 2).

## 4. Discussion

ERAS is a multimodal and multilevel approach in the management of surgical patients. The results on the clinical outcome of ERAS in terms of safety and reduction in complications are well-defined and reported in the literature [6,7,8,10,11]. The application of ERAS protocols needs the coordination and integration of different healthcare professionals and the active participation and compliance of the patient. ERAS programs involve the preoperative preparation of the patient for surgical procedures, both clinically and psychologically, with a dedicated communication strategy, systematic use of minimally invasive surgery, application of postoperative protocols, making the postsurgical course faster and more secure, and dedicated telemedicine platforms.

Application and implementation of ERAS protocols require additional costs in terms of dedicated healthcare professionals and telemedicine platforms. A recent systematic review about ERAS in colorectal surgery concluded that, ultimately, the greatest costs were attributed to personnel [16,17].

Our analysis does not focus much on the decrease in the costs related to the reduction in complications, but it tries to correlate the decrease in LOS with ERAS programs to a relevant advantage in terms of days of hospitalization gained and a greater number of treated patients. According to the literature [16,17,18], we suppose that ERAS protocol application implies a 50% reduction in average hospital stay. In our experience, we are recruiting patients for the TASED (Tele-Assistance Safe Early Discharge) study, aiming at analyzing the feasibility and patients’ compliance for discharge on postoperative day 3 after colonic resection. From January 2025, 24 patients submitted to colonic resection in our unit were enrolled in the ERAS program, with a rate of compliance to protocol of 87.5% (21/24). The mean LOS of our initial experience with ERAS protocol in the subgroup of patients with colonic resection was 3 days (range 3–15 days), comparable to the data in the literature and to the data estimated in the study (LOS reduction of 50%). All the patients enrolled were mobilized in I GPO, resumed valid diuresis in I GPO, and 95.5% of patients resumed oral alimentation in I GPO. We registered a 12.5% rate of overall complications and no readmission.

As reported in other publications [16,17,18], cost analysis of ERAS programs must consider benefits in terms of days of hospitalization gained and improvement in the turnover of the patients treated. Our study confirms this setting also for major surgical procedures in second-level (spoke) centers. Interestingly, in exchange for an investment lower than 1 million euros, it confirms an average revenue–cost ratio up to 2.92 (Scenario B), also in the unusual situation of a post-pandemic phase needing a national waiting list management planning (PNGLA, “Piano nazionale di gestione delle liste d’attesa”) [State-Regions Statement February 2019] (Table 2). Undoubtedly, this suggestion from the data needs an assumption. The large-scale concretization of these results should necessarily account for the diffusion of at-home support for patients, allowing an efficient and effective rehabilitation phase.

The main limit of our study is that it is based on an estimation of the economic impact produced by the hypothetical and not realistic application of an ERAS program in four models, in four hypothetical scenarios. Moreover, it must be pointed out that no subgroup analysis was conducted in the two surgical groups of study (colon resection for colorectal cancer and hip replacement), making it impossible to clarify the differences in the cost–effectiveness of ERAS across different types of surgery.

Another controversial issue of our analysis is that the presumed advantages in the application of ERAS protocols may be lower than the theoretical value. In fact, a growing turnover of patients cannot be guaranteed only by shortening the length of hospital stay but also by some other factors, such as operating room capacity and healthcare staff availability.

Finally, the analysis did not involve the costs of outpatient healthcare resources, even though they are highly relevant for achieving favorable outcomes.

Furthermore, if the price of the material for telemedicine is higher in the beginning, then it is just the consumable, so if we analyze a longer period of time, the cost–effectiveness ratio should be bigger than expected.

## 5. Conclusions

Our study confirms that the implementation of the ERAS programs is feasible even in second-level centers with fewer financial resources available. ERAS, in a directly proportional way with compliance to protocol, leads to a decrease in LOS, costs of hospitalization, and resources’ utilization as described in the literature [18], thus increasing the volume of patients treated per year and reducing the need for additional hospitalization from the waiting list.

In addition to the better clinical outcomes of ERAS programs, it should be emphasized that the application of ERAS protocols could be particularly functional and strategic in health policy and should be currently recommended in every hospital and healthcare system to improve sustainability.

## Figures and Tables

**Table 1:** Impact of ERAS on LOS in one year.

Surgical Procedures	# Procedures September 2022–October 2023	Length of Stay (Days) September 2022–October 2023		Annual LOS Estimation with ERAS (Days)
		Total Days	Mean	
Colon resection for tumors	37	312	8.43	156
Hip replacement	150	1.289	8.59	644.5
Total	187	1.601	8.56	800.5

#: Number of procedures

**Table 2:** Impact of ERAS on revenues and hospitalization.

	Hypotheses			Results			
Scenario	ERAS Coefficient of Application	Procedures on New Hospitalizations	Additional Hospitalization Days/Year	Additional Hospitalization/Year	Additional Procedure/Week	Δ (Additional Revenues—Additional Costs)	
						Δ	Ratio
A	100%	Overall	800.5	151	3.43	+424,705.83	2.50
B	100%	Only PNGLA	800.5	197	4.48	+543,411.28	2.92
C	50%	Overall	400.25	65	1.48	+31,858.70	1.11
D	50%	Only PNGLA	800.5	94	2.14	+110,431.83	1.39

## Data Availability

The original contributions presented in this study are included in the article. Further inquiries can be directed to the corresponding author.
